# Comparative efficacy and safety of Chinese herbal medicine for knee osteoarthritis: A protocol for systematic review and network meta-analysis

**DOI:** 10.1097/MD.0000000000026671

**Published:** 2021-07-23

**Authors:** Lei Yang, Bo-yu Wu, Lu Ma, Zheng-dong Li, Hui Xiong

**Affiliations:** Hunan University of Chinese Medicine, Changsha, China.

**Keywords:** Chinese herbal medicine, knee osteoarthritis, network meta-analysis

## Abstract

**Background:**

Knee osteoarthritis (OA) is a major public health concern causing chronic disability as well as a substantial burden on health care and the economy. However, effective treatments for knee OA were still not available. Numerous clinical studies have suggested that Chinese herbal medicine (CHM) seems to be clinically effective in treating knee OA. Thus, this study aims to evaluate the efficacy and safety of CHM in the treatment of knee OA through a systematic review and network meta-analysis.

**Methods:**

A comprehensive search will be performed in PubMed, Cochrane Library, Embase, Web of Science, China National Knowledge Infrastructure, VIP Database, Wanfang Database, Chinese Biomedical Database, and 3 clinical trials registration websites, from the database inception to May 2021. Randomized controlled trials meeting the eligible criteria based on the PICOS framework will be included. All studies fulfilling the eligible criteria will be assessed for risk of bias using the Cochrane Collaboration's tool. The primary outcome will be the visual analog scale (VAS), Western Ontario and McMaster Universities Osteoarthritis Index, and total effective rate. The secondary outcome is the incidence of adverse events. Data analysis will be performed using Stata, Addis, and WinBUGS.

**Discussion:**

This study will provide a reliable evidence to assess effectiveness and safety of CHM for knee OA, which may provide guidance for clinical practice.

**Systematic review registration:**

This study protocol has been registered on INPLASY202160060.

## 1 Introduction

Knee osteoarthritis (OA) is the most common chronic degenerative joint disease with a high incidence among the elderly worldwide.^[1,2]^ Clinically, knee OA patients suffer from slowly developing chronic joint pain, swelling, stiffness, limited range of motion, and deformity.^[3]^ It severely impacts the health and quality of life of sufferers and also leaves a tremendous economic and social burden.^[4]^ Present therapies for knee OA are mainly palliative, aiming to alleviate symptoms by reducing joint pain and improving motor function.^[5]^ According to international guidelines,^[6–8]^ the presently recommended pharmacological treatment options for knee OA include nonsteroidal anti-inflammatory drugs, analgesics, low potency opioids, and intraarticular corticosteroids, among others. Pharmacotherapies have a certain curative effect on relieving symptoms, but most of the drug are generally accompanied by adverse effects, including gastrointestinal disorders, circulatory system disorders, and risk of dependence or addiction.^[9,10]^ Due to the limited treatment regimens available, interest in complementary and alternative medicines is increasing worldwide.^[11,12]^

Chinese herbal medicine (CHM) is an important component of traditional Chinese medicine (TCM), and has been widely used for knee OA in China.^[11–14]^ At present, along with the introduction of evidence-based medical conception, many randomized controlled trials (RCTs) and systematic reviews have been conducted to assess the safety and efficacy of diverse CHM on knee OA, and the results were promising.^[12,13,15]^ However, most studies have focused on comparing CHM alone or CHM combined with conventional treatments versus conventional treatments or placebo. This reflects the absence of direct comparisons between diverse CHM therapy, and hence uncertainty regarding the comparative safety and efficacy among diverse CHM therapy. Moreover, there have been no systematic reviews or network meta-analyses (NMA) to date comparing different CHM therapy in the management of knee OA.

To address this issue, we will conduct a NMA, a method that allows simultaneous comparison of multiple treatments within a single meta-analysis.^[16]^ This study aims to compare the efficacy and safety of different CHM therapy for knee OA through a systematic review and NMA, incorporating evidence from RCTs.

## 2 Methods

This systematic review and NMA protocol follows the Preferred Reporting Items for Systematic Review and Meta-Analysis Protocols (PRISMA-P) guidelines.^[17]^ The protocol has been registered at INPLASY under the code INPLASY202160060, available at: https://inplasy.com/inplasy-2021-6-0060/

### 2.1 Eligible criteria

#### 2.1.1 Types of studies

RCTs that assessed the efficacy and safety of CHM for knee OA will be included. Languages will be restricted to English and Chinese. Descriptive studies, reviews, letters, conference abstracts, retrospective clinical studies, case reports, case series, protocols, animal studies, reports with incomplete data, studies unrelated to CHM, and knee OA will be excluded. For duplicate studies, the most informative and complete report will be selected.

#### 2.1.2 Types of participants

Participants (18 years or older) were diagnosed with knee OA based on radiographic evidence and clinical criteria.

#### 2.1.3 Type of interventions and comparisons

In the experimental group, any form of CHM will be included, including Chinese patent medicine, TCM decoction (eg, Duhuo Jisheng Decoction [15]), pills, among others. Considering that clinicians may combine CHM with conventional pharmacotherapy (western medicine), those studies will also be included. Patients in the control group were treated with conventional pharmacotherapy (western medicine) or placebo. In addition, we will exclude studies involving combination treatment of multiple CHM.

#### 2.1.4 Types of outcomes

The primary outcomes will include visual analog scale, Western Ontario and McMaster Universities Osteoarthritis Index, and total effective rate. The adverse events will be selected as a secondary outcome.

### 2.2 Search strategy

We will perform a comprehensive search of PubMed, Cochrane Library, Embase, Web of Science, China National Knowledge Infrastructure, VIP Database, Wanfang Database, and Chinese Biomedical Database, from their inceptions to May 2021. In addition, we will also search clinical trials registries (Clinicaltrials.gov, Chinese Clinical Trial Registry, and International Clinical Trials Registry Platform) for any missed RCTs. Search terms include knee OA, CHM, RCTs, and their synonyms. Our strategy will combine Medical Subject Heading with free-text terms. The specific search strategies will be adapted for each database. The detailed search strategy of PubMed is given in Appendix S1.

### 2.3 Study selection

**Figure 1:**
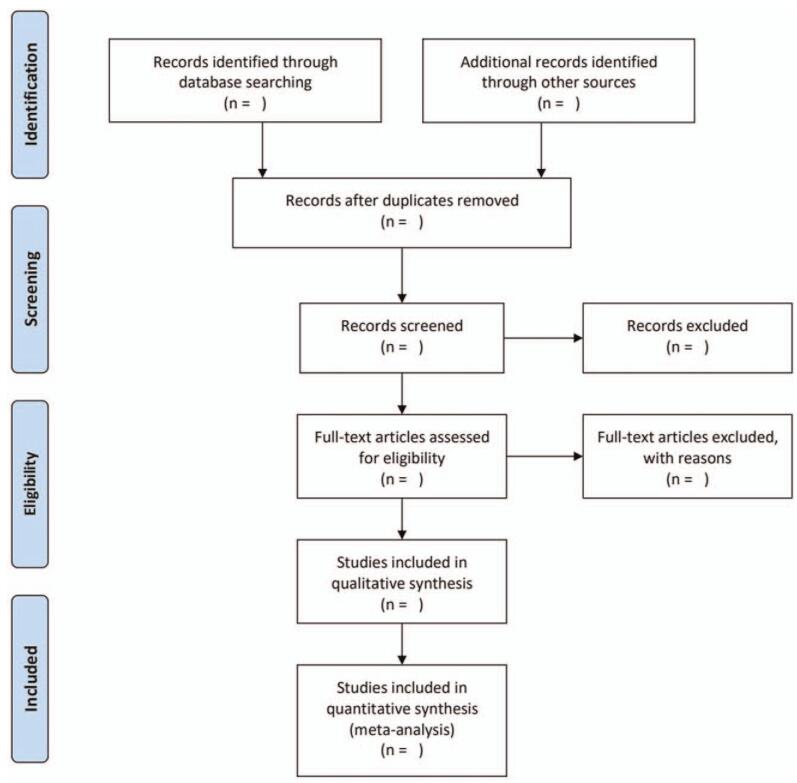
PRISMA flow diagram of the study selection process.

All studies retrieved from databases will be imported into the EndNote X8 software. After removing duplications, 2 reviewers will independently screen the title and abstracts of all studies. Then, full-text articles of potentially eligible studies will be screened for further assessment. Any discrepancies between the 2 reviewers will be resolved by a third researcher. A PRISMA flow diagram will be used to summarize the results of the whole selection process (Fig. 1).

### 2.4 Data extraction

Two reviewers will independently extract data using a standardized data extraction sheet. The following items will be extracted: first author, publication year, country, sample size, age and sex of participants, intervention details, treatment duration, follow-up period, and outcomes. We will try to contact corresponding authors for further information if important data are not reported in articles. Any disagreements will be solved in consultation with a third researcher.

### 2.5 Risk of bias assessment

For each included study, methodological quality will be assessed independently by two reviewers using the Cochrane Collaboration's tool for assessing risk of bias in RCTs.^[18]^ This risk-of-bias tool consists of 6 major domains of bias: selection bias, performance bias, detection bias, attrition bias, reporting bias, and other bias. Each domain will be categorized as low risk, high risk, and unclear risk. Also, a third reviewer will be available to resolve any disagreement.

### 2.6 Data analyses

#### 2.6.1 Pairwise meta-analysis

We will perform the pairwise meta-analysis with STATA 15.0. For dichotomous variables, outcomes will be expressed as odds ratio with 95% confidence intervals (CI), while for continuous variables, mean difference or standard mean difference with 95% CI will be calculated. Heterogeneity between the studies will be assessed with the I-square (*I*^2^) statistic.^[19]^ A fixed-effect model will be selected when *I*^2^ <50%; otherwise, a random-effect model will be selected when *I*^2^ > 50%.

#### 2.6.2 Network meta-analysis

We will perform the NMA with Addis1.16.8, WinBUGS 1.4.3, and STATA 15.0. A random effects model will be employed because of anticipated heterogeneity. The outcomes of dichotomous variables or continuous variables will be estimated by odds ratio, mean difference, and standard mean difference with their 95% CI respectively. The Brooks-Gelman-Rubin method will be used to assess the convergence of iterations. Convergence will be calculated using the Potential Scale Reduction Factor, with Potential Scale Reduction Factor closed to 1 indicating a better convergence.^[20]^ We will use node-splitting method to explore the inconsistency between direct and indirect evidence. Besides, the surface under the cumulative ranking curve will be applied to rank the size effect of treatments.^[21]^

#### 2.6.3 Subgroup and sensitivity analyses

If the heterogeneity or inconsistency among the included studies is detected, a subgroup analysis will be performed. Subgroup analysis will be conducted according to sample size, types of conventional pharmacotherapy (western medicine), treatment duration, length follow-up, and other relevant parameters. If feasible, we will perform a sensitivity analysis to explore the stability of the results. The influence of each study on the overall effect will be analyzed by removing one study at a time.

#### 2.6.4 Publication bias

To assess publication bias, we will apply the comparison-adjusted funnel plot to detect the effects of small studies.^[22]^ The Egger test will be used to evaluate funnel plot asymmetry.^[23]^

### 2.7 Quality of evidence

The present Grades of Recommendations Assessment Development and Evaluation (GRADE) guidance will be used to assess the quality of evidence.^[24]^ We will allocate the quality of the evidence as high, moderate, low, or very low according to the GRADE guidance.

### 2.8 Ethics and dissemination

Given that the protocol does not involve the collection of private information or affect the patient's right, so this study does not require ethical approval.

## 3 Discussion

Knee OA is a prevalent degenerative osteoarticular disease characterized by progressive destruction of articular cartilage, which lead to impaired physical function and decreased quality of life.^[1,2,3,25]^ Although there are symptomatic treatments for knee OA patients, presently there is no effective approaches to prevent or cure knee OA.^[26]^ Owing to this unsatisfactory status quo, complementary and alternative medicines have recently received increasing attention from researchers.^[11,12]^. CHM is the main method of complementary and alternative medicines, and the recent studies based on the effects of CHM have generally highlighted its effectiveness.^[11–15,25,27]^ However, most studies focus on the effectiveness of single CHM therapy. A direct comparison between the CHM therapies is lacking and it remains uncertain which CHM therapy are the most effective and safest for the management of knee OA. Therefore, we will perform a NMA to address this issue as well as provide a rank of various CHM therapy. We anticipate the following limitations. First, we will only include RCTs written in English and Chinese, it may cause a potential bias. Besides, treatment frequency and follow-up time used across studies could be the source of heterogeneity. In this case, we will further conduct a subgroup analysis if permitting. The results of this protocol will be published in an international peer-reviewed journal, it will find out which CHM therapy has the best efficacy and safety in knee OA. We will update the protocol when supplements are required.

## Author contributions

**Data curation:** Lei Yang, Bo-yu Wu, Hui Xiong.

**Formal analysis:** Lei Yang, Bo-yu Wu, Hui Xiong.

**Funding acquisition:** Lei Yang, Lu Ma.

**Investigation:** Bo-yu Wu, Lu Ma, Hui Xiong.

**Methodology:** Lu Ma, Zheng-dong Li, Hui Xiong.

**Project administration:** Lei Yang, Bo yu Wu, Lu Ma.

**Resources:** Lei Yang, Bo-yu Wu, Hui Xiong.

**Software:** Bo yu Wu, Lu Ma.

**Supervision:** Hui Xiong.

**Writing – original draft:** Lei Yang, Bo yu Wu.

**Writing – review & editing:** Hui Xiong.

## Supplementary Material

Supplemental Digital Content
