# Supplementary material for: Comparative efficacy and safety of Chinese herbal medicine for knee osteoarthritis: A protocol for systematic review and network meta-analysis
Source: Medicine (Baltimore). 2021 Jul 23;100(29):e26671. doi: 10.1097/MD.0000000000026671 (PMC8294919; doi:10.1097/MD.0000000000026671)
Supplement: Supplemental Digital Content [file medi-100-e26671-s001.docx]

| #ID | Topic or intervention | Query |
| --- | --- | --- |
| #1 | Disease | ("Osteoarthritis, Knee"[Mesh]) OR ("Osteoarthritis"[Mesh] AND "Knee  Joint"[Mesh]) OR ("Osteoarthritis"[Mesh] AND (Knee[Title/Abstract] OR  Knees[Title/Abstract])) OR ((Osteoarthritis[Title/Abstract] OR  Osteoarthritides[Title/Abstract] OR Arthritis[Title/Abstract] OR  Arthritides[Title/Abstract]) AND "Knee Joint"[Mesh]) OR ("Knee  Osteoarthritis"[Title/Abstract] OR "Knee Osteoarthritides"[Title/Abstract]  OR "Osteoarthritis of Knee"[Title/Abstract] OR "Osteoarthritis of  Knees"[Title/Abstract] OR "Knee OA"[Title/Abstract] OR "Knees  OA"[Title/Abstract]) OR ((Osteoarthritis[Title/Abstract] OR  Osteoarthritides[Title/Abstract] OR Arthritis[Title/Abstract] OR  Arthritides[Title/Abstract]) AND (Knee[Title/Abstract] OR  Knees[Title/Abstract])) |
| #2 | intervention | "Herbal Medicine"[Mesh] OR "Drugs, Chinese Herbal"[Mesh] OR "Plants, Medicinal"[Mesh] OR "Phytotherapy"[Mesh] OR "Medicine, Traditional"[Mesh] OR herb[Title/Abstract] OR herbal[Title/Abstract] OR plant[Title/Abstract] OR plants[Title/Abstract] OR phytotherapy[Title/Abstract] OR "Medicine, Traditional"[Title/Abstract] |
| #3 | Study design | "Randomized Controlled Trial" [Publication Type] OR  Randomized[Title/Abstract] OR Randomised[Title/Abstract] OR  Randomization[Title/Abstract] OR Randomisation[Title/Abstract] |
| #4 | Final query | #1 AND #2 AND #3 |
